# CNN-LRP: Understanding Convolutional Neural Networks Performance for Target Recognition in SAR Images

**DOI:** 10.3390/s21134536

**Published:** 2021-07-01

**Authors:** Bo Zang, Linlin Ding, Zhenpeng Feng, Mingzhe Zhu, Tao Lei, Mengdao Xing, Xianda Zhou

**Affiliations:** 1 School of Electronic Engineering, Xidian University, Xi’an 710071, China; bzang@mail.xidian.edu.cn (B.Z.); llding@stu.xidian.edu.cn (L.D.); zhumz@mail.xidian.edu.cn (M.Z.); xmd@xidian.edu.cn (M.X.); 2 School of Electronic Information and Artificial Intelligence, Shaanxi University of Science and Technology, Xi’an 710021, China; leitaoly@163.com; 3 Beijing Aerospace Automatic Control Institute, Beijing 100070, China; zhouxianda999@gmail.com

**Keywords:** synthetic aperture radar (SAR), target recognition, layer-wise relevance propagation (LRP), convolutional neural networks (CNN) understanding

## Abstract

Target recognition is one of the most challenging tasks in synthetic aperture radar (SAR) image processing since it is highly affected by a series of pre-processing techniques which usually require sophisticated manipulation for different data and consume huge calculation resources. To alleviate this limitation, numerous deep-learning based target recognition methods are proposed, particularly combined with convolutional neural network (CNN) due to its strong capability of data abstraction and end-to-end structure. In this case, although complex pre-processing can be avoided, the inner mechanism of CNN is still unclear. Such a “black box” only tells a result but not what CNN learned from the input data, thus it is difficult for researchers to further analyze the causes of errors. Layer-wise relevance propagation (LRP) is a prevalent pixel-level rearrangement algorithm to visualize neural networks’ inner mechanism. LRP is usually applied in sparse auto-encoder with only fully-connected layers rather than CNN, but such network structure usually obtains much lower recognition accuracy than CNN. In this paper, we propose a novel LRP algorithm particularly designed for understanding CNN’s performance on SAR image target recognition. We provide a concise form of the correlation between output of a layer and weights of the next layer in CNNs. The proposed method can provide positive and negative contributions in input SAR images for CNN’s classification, viewed as a clear visual understanding of CNN’s recognition mechanism. Numerous experimental results demonstrate the proposed method outperforms common LRP.

## 1. Introduction

Synthetic aperture radar (SAR) can generate radar images with both high range-resolution and Doppler-resolution by synthesizing a series of small aperture antennas into an equivalent large aperture antenna. SAR can work in various extreme conditions, e.g., mist, rain, clouds, etc., thus it is widely applied in electronic reconnaissance, topographic mapping, and vehicle surveillance [1,2]. Although numerous SAR images have been generated, the interpretation of SAR images develops far behind imaging them. In SAR image interpretation, target recognition is usually regarded as one of the most challenging tasks [1,3]. Generally, target recognition can be compartmentalized into two steps: First, some pre-processing techniques will be performed on raw SAR images, such as filtering, edge detection, region of interest (ROI) extraction, and feature extraction. Second, a classifier is used to categorize them to their corresponding class according to the divergence among extracted features [4,5]. However, such complex individual procedures usually bring a huge computation burden, causing difficulty in realizing real-time application and device miniaturization.

To alleviate these limitations of traditional methods, numerous deep learning based target recognition algorithms were proposed in recent decades, particularly combined with convolutional neural network (CNN). CNN is an end-to-end structure which requires no pre-processing implementation [6]. The input data are abstracted as discriminative features by different convolutional units (kernel, filter, channel) in deep layers for classification. Convolutional units cannot only reduce the number of trainable parameters but also preserve local characteristics in neighbor regions in images. In SAR image processing, much related work has been studied and achieved amazing performance. Wu et al. adopted CNN as a classifier in target recognition and achieved higher recognition rate than an SVM in [7]. Zhang et al. proposed a fast training method for SAR large scale samples based on CNN for targets recognition in [3], which can effectively reduce over-fitting. Zhou et al. proposed a large-margin softmax batch-normalization CNN (LM-BN-CNN) for SAR target recognition in [8], which simultaneously obtained the superior accuracy and convergence speed compared with other general CNN structures. Zhang et al. proposed a feature fusion framework (FEC) based on scattering center features and deep CNN features which achieves superior effectiveness and robustness under both standard operating conditions and extended operating conditions [9]. Oh et al. proposed a CNN-based SAR target recognition network with pose angle marginalization learning which outperforms the other state-of-the-art SAR-ATR algorithms, yielding the correct target recognition rate with an average of 99.6% [10].

Although the recognition accuracy is increasingly higher in aforementioned deep learning methods, CNN is usually used as a “black box” since the inner recognition mechanism of CNN is still opaque. Specifically, the semantic information of features extracted by the deep convolutional layers is often difficult for humans to understand [11,12]. In this case, the reliability of recognition results is less convincing compared with traditional methods. On the other hand, unexplainability of CNN also makes it difficult to analyze the causes of wrong results. To provide a reasonable explanation of “black box”, many scholars obtained some meaningful achievements. Some of them explain neural network from perspective of structure. Setzu et al. proposed GLocalX to generalize local explanations expressed in form of local decision rules to global explanations iteratively by aggregating them hierarchically [13]. Xiong et al. proposed a totally interpretable CNN, SPB-Net, by deep unfolding to suppress speckles in SAR images [14]. In comparison, another group of researchers attempt to visualize what CNN learns from input data [15,16,17,18,19], mainly divided into three categories: perturbation methods, activation methods and propagation methods. The former two methods highlight the regions of the input image that are responsible for CNN’s correct classification, while the latter can further detect the regions that are negative for CNN’s judgment in addition. Perturbation methods usually occlude the input image with a sliding patch to check whether the occluded region can cause a dramatic drop of recognition accuracy. Perturbation methods are intuitive and easy to implement; however, they have two obvious limitations: (1).The computation burden is huge for this traversal search. (2). Different data may require specifically designed occlusion rules, leading to huge cost of algorithm design. Perturbation methods are seldom directly adopted to generate heatmaps; instead, they are usually used to verify the performance of other visualization methods. Activation methods visualize CNN decisions by artfully combining the feature maps in deep convolution layers. These kinds of methods integrate input image, features in deep layers and final output of CNN, which obtained remarkable and amazing achievements [16,17,18,19]. However, in some scenarios, it is not enough to know which parts of the input images are responsible for CNN’s recognition. We also need to know, more specifically, which parts contribute positively to recognition and which parts contribute negatively. Propagation methods can solve this problem well [20]. They are a kind of pixel rearrangement methods which propagates CNN’s output backward to input space layer for layer. Amin et al. combined layer-wise relevance propagation (LRP) and sparse auto-encoder to obtain an understanding of CNN’s performance on radar-based human motion recognition [21]. However, the auto-encoder only contains fully-connected layers. For SAR images, such a simple structure is not powerful enough to extract the features which can achieve high recognition accuracy. Therefore, we propose a novel LRP method particularly designed for CNN’s performance in SAR image target recognition. In our proposed method, we provide a concise form of the correlation between the output of convolutional layer and weights of convolutional units.

The contributions of this paper can be summarized as: (1) To the best of our knowledge, this is the first time LRP and CNN are combined in SAR image interpretation; (2) In comparison to [21], the proposed method can provide the positive and negative contributions under much higher recognition accuracy.

The remainder of this paper is organized as follows. For a comprehensive understanding of propagation methods, Section 2 reviews basic LRP. Section 3 introduces the the proposed method in detail. Section 4 provides numerous experimental results from various perspectives to compare the performance of the proposed method with basic LRP. Section 5 discusses the experimental results and clarifies some confusion.

## 2. Principle of Layer-Wise Relevance Propagation

In this section, we take an application of LRP approach combined with sparse encoder in understanding of human motion radar signals as an example to introduce its principle. Section 2.1 concisely describes sparse auto-encoder and Section 2.2 introduces how LRP works in this structure.

### 2.1. Sparse Autoencoder

**Figure 1:**
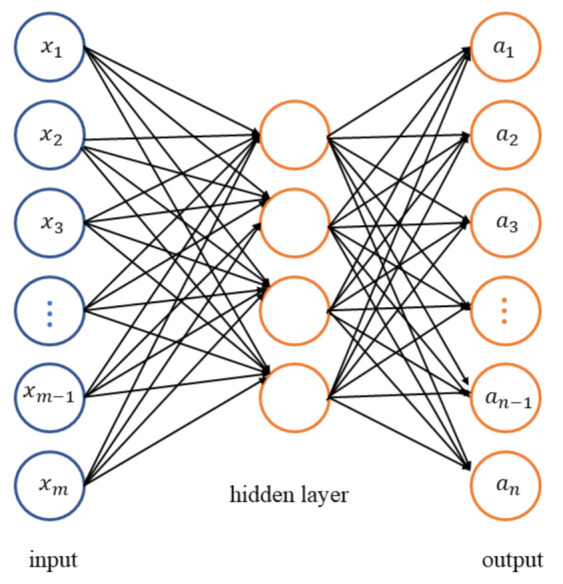
Structure of sparse auto-encoder.

Sparse auto-encoder is a kind of fully-connected neural network with symmetrical structure. Sparse auto-encoder can be divided into encoder and decoder. The encoder attempts to obtain the sparse representation of the input data via a single hidden layer that typically has fewer neurons than the input while the decoder has the same number of neurons with the input data, as shown in Figure 1. The loss function of sparse auto-encoder is a minimization of measurement between output of network $a_{n}$ and original input image $x_{m}$. Once the sparse auto-encoder is well trained, the output of hidden layer can be deemed as a discriminative representation of input data.

**Figure 2:**
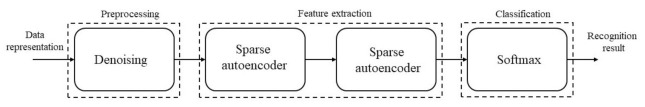
Deep learning based architecture for target recognition.

The neurons between layers are connected by the weights and biases. Sigmoid function σ(·) is adopted as activation to weighted and biased input data units $x_{m}$, i.e., the output of the hidden layer *n*th neuron is

(1) $$\begin{array}{c} a_{n}=\sigma \left(\sum_{m}x_{m}\omega_{m,n}+b_{n}\right) \end{array}$$

where $w_{m,n}$ and $b_{n}$ denote the weight and the bias, respectively. $w_{m,n}$ and $b_{n}$ are two trainable parameters learned by minimizing the aforementioned cost function. When the auto-encoder is well trained (the output of encoder can be deemed as representative and sparse features of the input data), a classifier with softmax regression can be performed following the encoder to categorize the input data into its most probable class, as shown in Figure 2. More details about the architecture can be found in [22].

### 2.2. Layer-Wise Relevance Propagation

Layer-wise relevance propagation (LRP) is a propagation-based explanation framework, which is applicable to general neural network structures, including deep neural networks, LSTMs, and Fisher vector classifiers. LRP explains individual decisions of a model by propagating the prediction from the output to the input using local redistribution rules [23]. The overall idea of LRP is to understand the contribution of a single pixel of an image *x* to the prediction f(x) made by a classifier *f* in an image classification task. Assume that the first layer of the neural network are the inputs, i.e., the original image, and the last layer is the real-valued prediction output of the classifier *f*. The contribution of *n*th neuron in the *l*th layer to the activation of the *m*th neuron in the next layer l+1 is modeled as a vector *z*:

(2) $$\begin{array}{c} z_{n,m}=a_{n}^{\left(l\right)}\omega_{n,m}^{\left(l,l+1\right)} \end{array}$$

LRP approach assumes that we have a relevance score $R^{\left(l\right)}$ when backward propagating from one layer to the next, i.e.,

(3) $$\begin{array}{c} f\left(x\right)=R^{\left(1\right)}=\cdots =R^{\left(l\right)}=R^{\left(l+1\right)} \end{array}$$

In fact, the classification function f(·) of the input image *x* can be deemed as the relevance of the last layer. Note that each neuron in a certain layer has corresponding relevance, thus the relevance $R^{\left(l\right)}$ in layer *l* is computed as the sum of relevance $r_{n}^{\left(l\right)}$ of all *N* neurons in layer *l*:

(4) $$\begin{array}{c} R^{\left(l\right)}=\sum_{n=1}^{N}r_{n}^{\left(l\right)} \end{array}$$

When the output of the neural network is propagated backward to the first layer, a heatmap $h=r_{n}^{\left(1\right)}$ is obtained as the following iteration:

(5) $$\begin{array}{c} r_{n}^{\left(l\right)}=\sum_{m}\left(\alpha \frac{z_{n,m}^{+}}{\sum_{n^{{}^{\prime }}}z_{n,m}^{+}}+\beta \frac{z_{n,m}^{-}}{\sum_{n^{{}^{\prime }}}z_{n,m}^{-}}\right)r_{m}^{\left(l+1\right)} \end{array}$$

where α+β=1, $z_{n,m}^{+}$ and $z_{n,m}^{-}$ are the positive and negative part of $z_{n,m}$, respectively.

**Figure 3:**
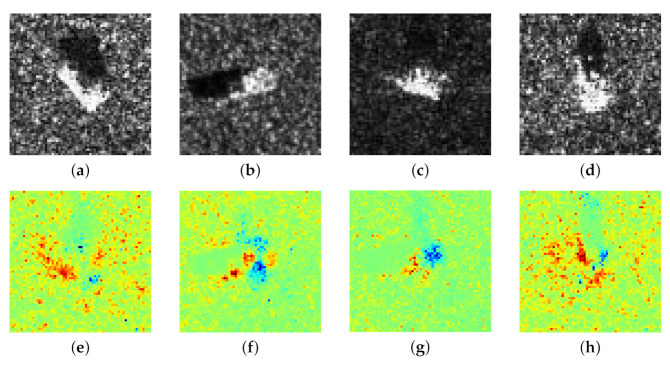
LRP heatmaps of SAR images. (**a**–**d**) are SAR images of 2S1 (self-propelled artillery), SN_9563 (armor vehicle), D7 (bull dozer), ZIL131 (cargo truck), and (**e**–**h**) are corresponding LRP heatmaps.

Equation (2) is a very concise iterative form and is friendly to implementation, whereas it is only available for neural networks with fully-connected layers, like sparse auto-encoder rather than CNN. For some images of simple objects, such as MINIST and spectrum data [21,24], sparse auto-encoder can extract features discriminative enough for classification; however, such structure is not powerful enough to process complex SAR images. The discrepancy between two classes of SAR images is not only different targets, but also may be related to scattering angle, medium density, interference, etc. [25,26]. This property of SAR images brings in two problems when sparse auto-encoder is adopted as a feature extractor: (1) The extracted features can only achieve low recognition accuracy; (2) The LRP heatmap shows puzzling regions of positive and negative contribution for classification. In fact, the heatmap *h* is closely related to the parameters of the network model, thus a wrong classification probably leads to an unreasonable heatmap. Here we exhibit several SAR images from MSTAR dataset (The detailed information of MSTAR is introduced in Section 4) and their corresponding LRP heatmaps in Figure 3. Apparently, it is confusing for people to understand these pixels are positive contributions and negative contributions, respectively.

## 3. Our Method

Different from fully-connected networks, CNN involves in weight sharing in convolutional layer and downsampling in pooling operation. Therefore, Equation (2) of common LRP can not be applied to CNN directly. Here we denote $\omega^{\left(l,l+1\right)}$ as the relationship of weight $\omega^{\left(l,l+1\right)}$ between *l*th layer and those of next layer (l+1)th layer. $\omega^{\left(l,l+1\right)}$ is in size of (N,C,M,M), where *N* and *C* are the number of convolutional kernels and channels of each kernel in the *l*th layer, respectively. *M* denotes the width and height of convolutional kernels in *l*th layer. $a^{\left(l\right)}$ is the output of the *l*th layer in size of (C,W,H). The specific relationship between contribution *z* and $a^{\left(l\right)}$ and $\omega^{\left(l,l+1\right)}$ is described as follows:

(6) $$\begin{array}{c} z\left[n,c,w,h\right]=a^{\left(l\right)}\left[c,m+W*\Delta ,m+H*\Delta \right]\omega^{\left(l,l+1\right)}\left[n,c,m,m\right] \end{array}$$

where (n,c,w,h) refers to the corresponding element of *z*, n=1,2,⋯,N, c=1,2,⋯,C, w=1,2,⋯,W, h=1,2,⋯,H, and m=1,2,⋯,M. In this case, the relevance $R^{\left(l\right)}$ can be calculated by Equations (4) and (5). It should be noted that the relevance map of the next layer needs to be upsampled to the output size of the upper convolutional layer due to pooling operation, which can be described as:

(7) $$\begin{array}{c} R_{Q}^{l}=S_{up}{\left(R^{l}\right)}_{Q} \end{array}$$

where $S_{up}{{(}^{.})}_{Q}$ means upsampling relevance maps to the size of the output of the upper convolutional layer (*Q*,*Q*).

**Figure 4:**
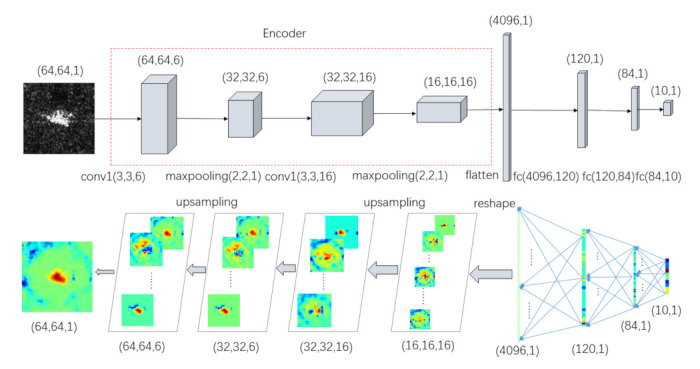
Flowchart of LRP method for CNN.

The details and flowchart of LRP method for CNN are described in Algorithm 1 and Figure 4.
**Algorithm 1** CNN-LRP**Input:** the original SAR image I in size of (1,W,H)
1:model f2:parameters: α, β3:**for***l* in [ L−1,...,1] **do**:4:    **if** *l* in classification layers **then**:5:             $z_{n,m}$ as in Equation (2)6:             $r_{n}^{\left(l\right)}$ as in Equation (5)7:             $R^{l}$ as in Equation (4)8:    **else** if *l* in convolution layers then:9:             z[n,c,w,h] as in Equation (6)10:             $r_{n}^{\left(l\right)}$ as in Equation (5)11:             $R^{l}$ as in Equation (4)12:    **end if**13:    **if** *l* in maxpooling layers **then**:14:              $R_{Q}^{l}$ as in Equation (7)15:    **end if**16:**end for**17:$H^{\left(\mathrm{LRP}\right)}$ = $R^{\left(1\right)}$
**Output:** the heatmap $H^{\left(\mathrm{LRP}\right)}$ in size of (1,W,H)


## 4. Experimental Results

In this section, we compare the performance of common LRP with sparse auto-encoder and the proposed method with CNN. Next, we analyze the results of our proposed method from several perspectives. The experimental dataset adopted in this paper is the real measured SAR images of ground stationary targets of 10 classes of vehicles, namely 2S1 (self-propelled artillery), BRDM_2 (armored reconnaissance vehicle), BTR60 (armored transport vehicle), D7 (bulldozer), T62 (tank), ZIL131 (cargo truck), ZSU234 (self-propelled anti-aircraft gun), and T72 (tank). High-resolution focused synthetic aperture radars with a resolution of 0.3 m × 0.3 m are used in this program, which work in the X-band, and the polarization mode is HH. For simplicity, we utilize a lightweight auto-encoder with only convolutional layers. Adaptive moment estimation (Adam) was adopted as the optimizer, learningrate = 1 × $10^{-3}$, β = (0.9,0.999), ϵ = 1 × $10^{-8}$, weight-decay = 0), as shown in Figure 4. Note that the gist of this paper is not to manipulate a CNN structure or obtain a set of parameters with high recognition accuracy, but to provide a visual understanding of CNN’s performance on SAR images. Some other state-of-the-art CNN models can probably achieve higher recognition accuracy, whereas such complex structures may be obstacles for understanding of CNN.

### 4.1. Comparison of the Proposed Method and Common LRP

**Figure 5:**
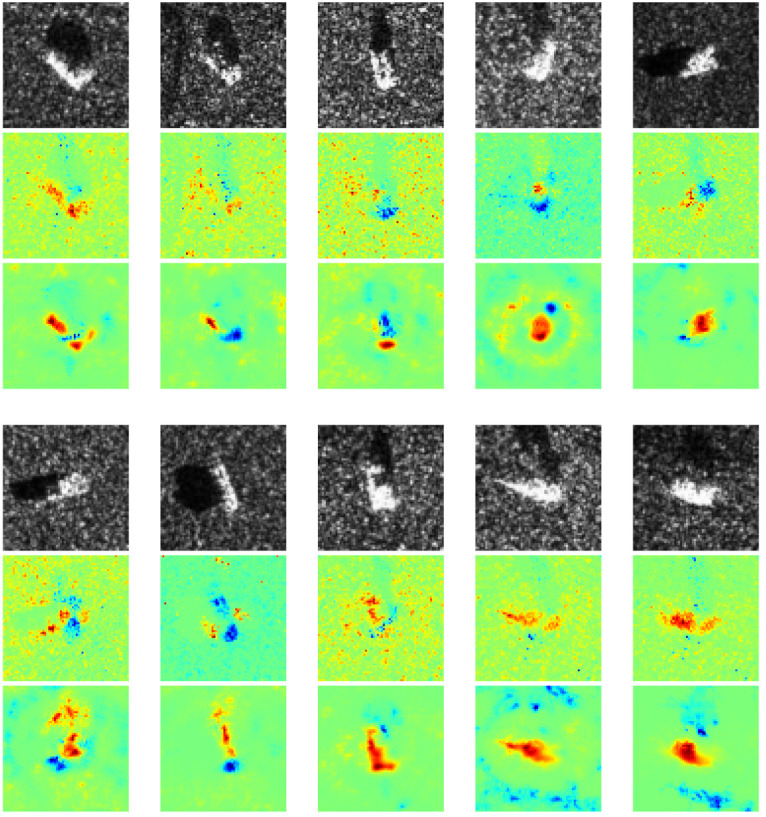
Comparison of common LRP and our method. The first and fourth row are the SAR images of ten classes: 2S1, BRDM_2, BTR_60, D7, SN_132, SN_9563, SN_C71, T62, ZIL131, and ZSU_23_4. The second and fifth row are corresponding heatmaps generated by common LRP. The third and sixth row are corresponding heatmaps generated by our method.

In this experiment, we apply our proposed method and common LRP in MSTAR dataset to obtain heatmaps. Figure 5 shows a SAR image from each class and their corresponding heatmaps generated by common LRP and our proposed method, respectively. In general, our proposed method can provide better interpretability of CNN than common LRP. Evidently, both positive and negative contributions in the heatmaps of common LRP are numerous scattered speckles, which is difficult to understand why CNN focuses on these elements. In contrast, our proposed method can provide more interpretable positive contributions which coincide with most parts of the target. Next, we will discuss the understanding of CNN’s classification by our method on several different cases.

### 4.2. Proposed Method versus Other Activation-Based Methods

**Figure 6:**
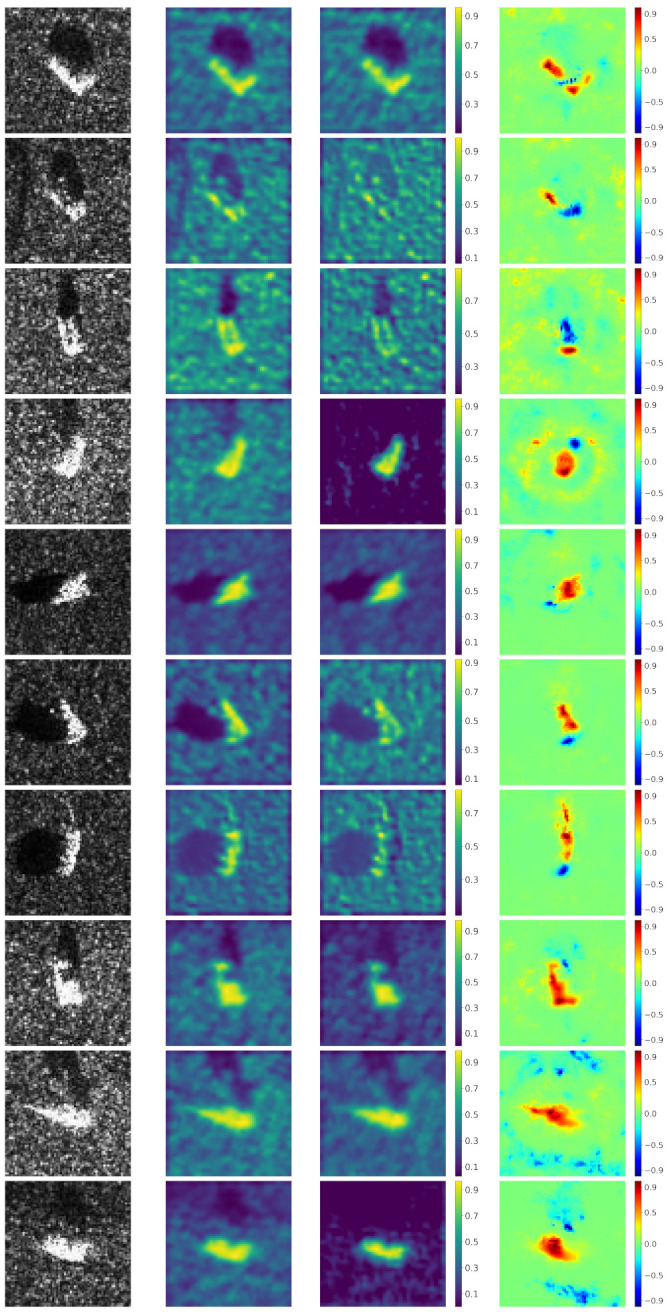
Comparison of Score-CAM, Ablation-CAM and our method. The first column is the SAR images of ten classes: 2S1, BRDM_2, BTR_60, D7, SN_132, SN_9563, SN_C71, T62, ZIL131, and ZSU_23_4. The second, third, and fourth columns are corresponding heatmaps generated by Score-CAM, Ablation-CAM and our method, respectively.

For CNN models, there have existed some activation-based methods, like various CAM methods. In CAM methods, the saliency heatmap $H^{CAM}$ is composed of linear weighted summation of feature maps in the last convolutional layer, defined as follows:

(8) $$\begin{array}{c} H^{CAM}=\sum_{k}\alpha_{c}^{k}A^{k} \end{array}$$

where $A^{k}$ is the *k*-th feature map in a convolutional layer, $\alpha_{c}^{k}$ denotes the weight of $A^{k}$ for the target class *c*. Saurabh Desai and Harish G. Ramaswamy proposed a Ablation CAM which uses the impact of each feature on CNN’s classification accuracy to formulate weights defined as:

(9) $$\begin{array}{c} \alpha^{ck\_Ablation}=\frac{S_{c}\left(A\right)-S_{c}\left(A\setminus A^{k}\right)}{S_{c}\left(A\right)}\; \end{array}$$

where $S_{c}\left(A\right)$ refers to the prediction score of class *c* when all the feature maps are sent to the classifier, and $S_{c}\left(A\setminus A^{k}\right)S_{c}\left(A\right)$ refers to the prediction score when a specific feature map $A^{k}$ is removed. Wang et al. proposed a Score CAM that takes the similarity between input image and each feature map as weights defined as:

(10) $$\begin{array}{c} \alpha^{ck\_Score}=C\left(A^{k}\right)=S_{c}\left(X\circ \Upsilon^{k}\right)-S_{c}\left(X_{b}\right)\; \end{array}$$

where $\Upsilon^{k}$ refers to the *k*-th feature map upsampled to the same size of the input image *X*, and $X_{b}$ is a baseline image which is usually set to 0. Here we also conduct these two CAM methods as comparison to our method. Nonetheless, it should be noted that LRP methods attempt to detect both positive and negative pixels influenced CNN’s classification, while CAM methods aim at providing a highlighted region which matches the target precisely, thus there are neither positive nor negative contribution in CAM heatmaps. To avoid confusion, we adopt different colormaps to exhibit LRP heatmaps and CAM heatmaps in Figure 6. Note that the value of elements in CAM heatmaps is normalized to [0,1], while the value is normalized to [−1,1] in LRP heatmaps. We can clearly observe that these two kinds of heatmaps reflect different information. CAM methods can highlight a region precisely matching the target’s shape but they can detect these pixels are positive or negative for CNN’s classification. In contrast, our method can vividly reflect both positive and negative pixels in input image for CNN’s classification.

### 4.3. Understanding of CNN from Different Perspectives

To understand CNN’s classification mechanism of SAR images, we categorized the heatmaps into three parts according to the distribution of positive and negative contributions. Specifically, the three categories are (1) positive and negative contributions are the targets, (2) positive contributions are targets while negative contributions are scattered speckles, and (3) negative contributions are targets while positive contributions are speckles.

**Figure 7:**
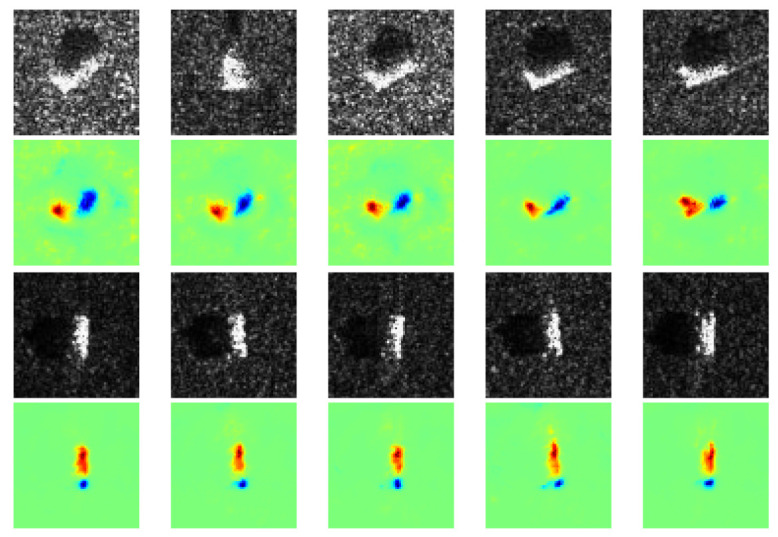
Positive and negative contributions are the targets. The first and third row are SAR images of two classes: 2S1 and SN_132. The second and fourth row are the corresponding heatmaps generated by our method.

We found that some heatmaps show both positive and negative contributions coincide with most parts of the target, as shown in Figure 7. Specifically, some parts of the target are conducive to CNN’s classification, while the rest are disturbing CNN’s classification. It is probably due to some discriminative components (positive contribution) of the target, like the barrel of self-propelled gun, and some confusing components (negative contribution) that all the vehicles own, like wheels. Besides, it can be observed the intra-class divergence of speckles is quite slight in a certain class, while the extra-class divergence is obviously tremendous. It indicates that for a specific class, the imaging conditions are the same, such as scattering angle, emission power, medium, etc., while for different classes, they are different. Therefore, the speckles make no contribution to classification, which matches human’s cognition.

**Figure 8:**
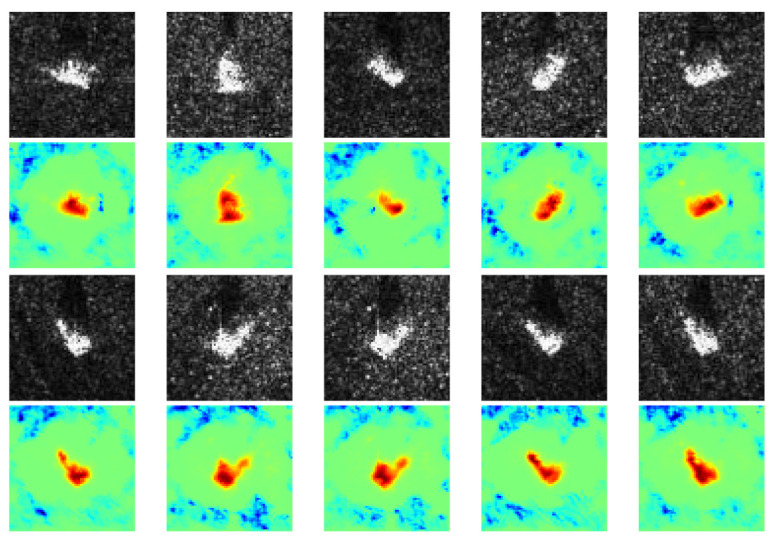
Positive contributions are targets while negative contributions are scattered speckles. The first and third rows are SAR images of two classes: D7 and ZSU_23_4. The second and fourth rows are the corresponding heatmaps generated by our method.

**Figure 9:**
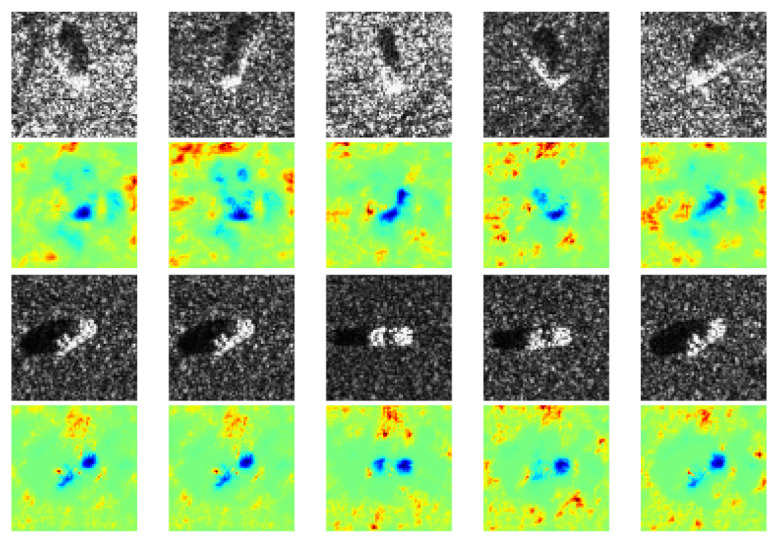
Negative contributions are targets while positive contributions are speckles. The first and third rows are SAR images of two classes: BRDM_2 and SN_C71. The second and fourth rows are the corresponding heatmaps generated by our method.

In contrast to the prior case, some other heatmaps show only positive contribution that coincides with the target, while negative contribution is located in some irregular areas, as shown in Figure 8. It is probably because imaging conditions of different classes are the same, thus similar interference speckles disturb the CNN’s classification. Conversely, some heatmaps exhibit native contribution which coincides with targets while positive contribution is located near speckles, as shown in Figure 9. It is probably because in these images, the targets are quite similar, whereas the speckles are the most discriminative features due to different imaging conditions.

## 5. Discussion

**Figure 10:**
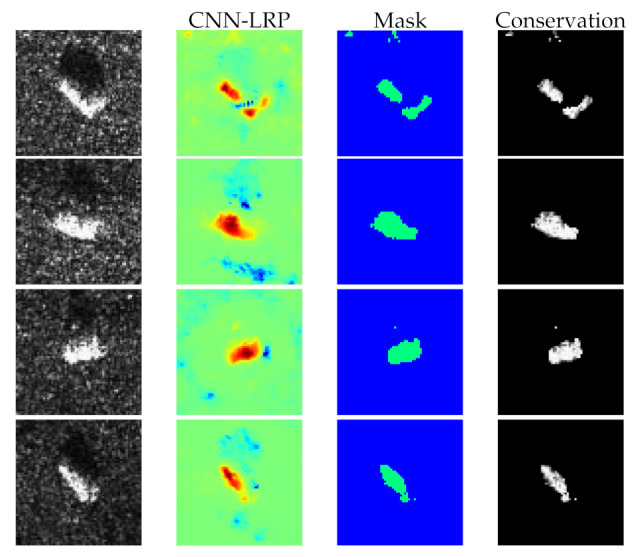
Results of conservation. The first column is SAR images of four classes: 2S1, ZSU_23_4, D7, T62. The second column is heatmaps generated by our method. The third column is binary mask. The fourth column is conservated images.

In this section, we will measure the qualitative performance of our method from classification accuracy. To further demonstrate the effectiveness of heatmaps, we binarize the heatmaps to obtain a set of masks by a threshold (in this experiment, we only preserve top 70% positive elements in heatmaps), thus a masked dataset can be generated by performing Hadmard product of masks and original data. This process can be viewed as filtering which only passes the positive or negative contribution pixels in the original SAR images. In this case, it means the preserved pixels really make positive contribution for CNN’s classification if the classification accuracy changes not obviously. We utilize the proposed method and common LRP to generate masked data, respectively. Figure 10 shows several classes of images, their corresponding heatmaps, masks, and masked images. Then original data and two kinds of masked data are used to train three CNNs with the same structure and parameters. Table 1 shows the top 5 recognition accuracy of three conditions when only positive contributions are preserved. Here we only select the top five recognition accuracy because a large number of misclassified samples in the other classes probably lead to inaccurate heatmaps which are negative for CNN’s understanding. It is apparent from Table 1 that CNN and the proposed method outperform the sparse auto-encoder and common LRP, respectively. Note that although the recognition accuracy of masked data generated by our method declines slightly than original data, the accuracy of our method (93.15%) is still higher than that of common LRP (83.99%) dramatically.

**Figure 11:**
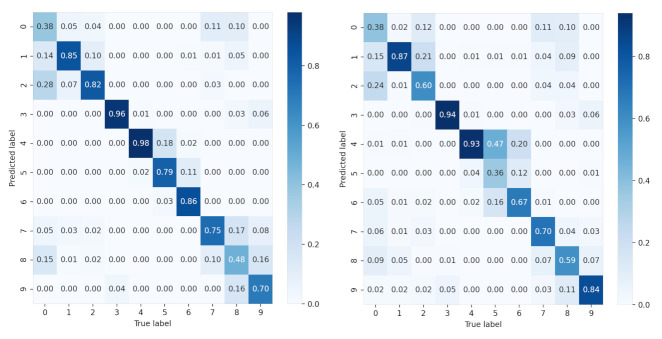
Confusion matrix of the sparse auto-encoder for 10 classes. The left is the confusion matrix of original data. The right is the confusion matrix of the data generated by common LRP.

**Figure 12:**
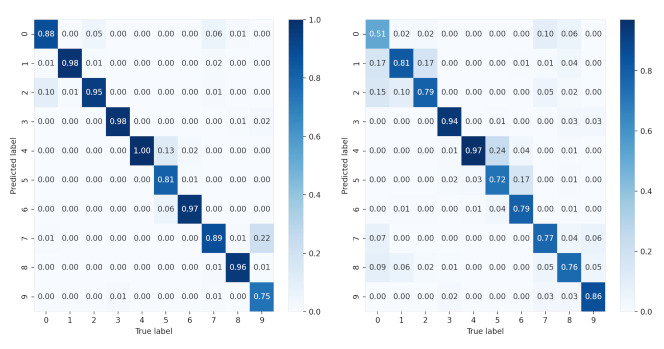
Confusion matrix of the CNN for 10 classes. The left is the confusion matrix of original data. The right is the confusion matrix of the data generated by our proposed method.

To further study the recognition accuracy of each class, we provide the confusion matrix of all SAR images of 10 classes under each condition in Figure 11 and Figure 12. It is clear that for sparse auto-encoder, misclassification occurs frequently among class 0, 1, 2, but seldom emerges when CNN and our proposed method are adopted.

## 6. Conclusions

In this paper, we proposed a new LRP method particularly designed for CNN’s classification in SAR image interpretation. Numerous experimental results on benchmark dataset MSTAR demonstrate our proposed method produces higher informative heatmaps that provide a visual understanding of the mechanism of CNN’s classification in comparison to common LRP on three cases: (1) Imaging conditions are corresponding to each class. In this case, both positive and negative contribution is located near some components of the target; (2) When the imaging conditions are similar for different classes, the speckles contribute negatively to classification; (3) When different classes of targets own similar shape or components, the target makes a negative contribution to classification. The results reveal that CNN indeed learns the most distinguishable information of different class to make the classification. In conclusion, the proposed method is an effective visualization tool of CNN’s inner mechanism and reveals that CNN’s mechanism matches human’s cognition. This finding may help to totally interpret the CNN to a “white box” in the future, which is our future research direction.

## Figures and Tables

**Table 1:** Recognition accuracy of positive contribution.

Sparse	Common LRP	CNN	Our Method
89.38%	83.99%	97.96%	93.15%

## Data Availability

In this paper, we adopted MSTAR dataset as the experimental dataset. MSTAR program was supported by the Defense Advanced Research Projects Agency (DARPA) in the mid-1990s. The MSTAR dataset contains 10 classes of SAR images of military vehicle which collected by high-resolution focused synthetic aperture radar, namely 2S1 (self-propelled artillery), BRDM_2 (armored reconnaissance vehicle), BTR60 (armored transport vehicle), D7 (bulldozer), T62 (tank), ZIL131 (cargo truck), ZSU234 (self-propelled anti-aircraft gun), and T72 (tank). The MSTAR dataset is fully open access. Readers can get it from authors. Readers can contact the corresponding author (Z.F.) for further information by email (zpfeng_1@stu.xidian.edu.cn) if there are any problems.

## References

[1] Wang Y.P., Zhang Y.B., Qu H.Q., Tian Q. Target Detection and Recognition Based on Convolutional Neural Network for SAR Image. Proceedings of the 2018 11th International Congress on Image and Signal Processing, BioMedical Engineering and Informatics (CISP-BMEI).

[2] Chao J.H., Park C.G. (2018). Multiple Feature Aggregation Using Convolutional Neural Networks for SAR Image-Based Automatic Target Recognition. IEEE Geosci. Remote Sens. Lett..

[3] Zhang Y., Song Y., Wang Y.P., Qu H.Q. A fast training method for SAR large scale samples based on CNN for targets recognition. Proceedings of the 2018 11th International Congress on Image and Signal Processing, BioMedical Engineering and Informatics (CISP-BMEI).

[4] Zhu M., Zhou X., Zang B. (2018). Micro-Doppler Feature Extraction of Inverse Synthetic Aperture Imaging Laser Radar Using Singular-Spectrum Analysis. Sensors.

[5] Zang B., Zhou X., Zhu M. (2019). Application of S-Transform Random Consistency in Inverse Synthetic Aperture Imaging Laser Radar Imaging. Appl. Sci..

[6] Chen L., Jiang X., Li Z. (2020). Feature-Enhanced Speckle Reduction via Low-Rank and Space-Angle Continuity for Circular SAR Target Recognition. IEEE Trans. Geosci. Remote Sens..

[7] Wu T.D., Yen Y., Wang J.H. Automatic Target Recognition in SAR Images Based on a Combination of CNN and SVM. Proceedings of the 2020 International Workshop on Electromagnetics: Applications and Student Innovation Competition (iWEM).

[8] Zhou F., Wang L., Bai X.R., Hui Y., Zhou Z. (2018). SAR ATR of Ground V ehicles Based on LM-BN-CNN. IEEE Trans. Geosci. Remote Sens..

[9] Zhang J., Xing M., Xie Y. (2020). FEC: A Feature Fusion Framework for SAR Target Recognition Based on Electromagnetic Scattering Features and Deep CNN Features. IEEE Trans. Geosci. Remote Sens..

[10] Oh J., Youm G.-Y., Kim M. (2021). SPAM-Net: A CNN-Based SAR Target Recognition Network With Pose Angle Marginalization Learning. IEEE Trans. Circuits Syst. Video Technol..

[11] Dong Y.P., Su H., Wu B.Y. Efficient Decision-based Black-box Adversarial Attacks on Face Recognition. Proceedings of the 2019 IEEE/CVF Conference on Computer Vision and Pattern Recognition (CVPR).

[12] Girshick R., Donahue J., Darrell T. Rich feature hierarchies for accurate object detection and semantic segmentation. Proceedings of the 2014 IEEE Conference on Computer Vision and Pattern Recognition (CVPR).

[13] Setzu M., Guidotti R., Monreale A., Turini F., Pedreschi D., Giannotti F. (2021). GLocalX - From Local to Global Explanations of Black Box AI Models. Artif. Intell..

[14] Xiong K., Zhao G., Wang Y., Shi G. (2021). SPB-Net: A Deep Network for SAR Imaging and Despeckling With DownSampled Data. IEEE Trans. Geosci. Remote Sens..

[15] Zhou B., Khosla K., Lapedriza A., Oliva A., Torralba A. Learning Deep Features for Discriminative Localization. Proceedings of the 2016 IEEE Conference on Computer Vision and Pattern Recognition (CVPR).

[16] Ramprasaath R.S., Michael C., Abhishek D. Grad-CAM: Visual Explanations from Deep Networks via Gradient-based Localization. Proceedings of the 2017 IEEE International Conference on Computer Vision (ICCV).

[17] Fu H.G., Hu Q.Y., Dong X.H., Guo Y.I., Gao Y.H., Li B. Axiom-based Grad-CAM: Towards Accurate Visualization and Explanation of CNNs. Proceedings of the 2020 31th British Machine Vision Conference (BMVC).

[18] Saurabh D., Harish G.R. Ablation-CAM: Visual Explanations for Deep Convolutional Network via Gradient-free Localization. Proceedings of the 2020 IEEE Winter Conference on Applications of Computer Vision (WACV).

[19] Feng Z., Zhu M., Stanković L., Ji H. (2021). Self-Matching CAM: A Novel Accurate Visual Explanation of CNNs for SAR Image Interpretation. Remote Sens..

[20] Bach S., Binder A., Montavon G., Klauschen F., Müller K.R., Samek W. (2014). On Pixel-Wise Explanations for Non-Linear Classifier Decisions by Layer-Wise Relevance Propagation. PLoS ONE.

[21] Amin M.G., Erol B. Understanding deep neural networks performance for radar-based human motion recognition. Proceedings of the 2018 IEEE Radar Conference (RadarConf18).

[22] Jokanovic B., Amin M., Ahmad F. Radar fall motion detection using deep learning. Proceedings of the 2016 IEEE Radar Conference (RadarConf16).

[23] Montavon G., Binder A., Lapuschkin S., Samek W., Müller K.R., Samek W., Montavon G., Vedaldi A., Hansen L., Müller K.R. (2019). Layer-Wise Relevance Propagation: An Overview. Explainable AI: Interpreting, Explaining and Visualizing Deep Learning.

[24] Montavon G., Samek W., Müller K.R. (2018). Methods for Interpreting and Understanding Deep Neural Networks. Digit. Signal Process..

[25] Ma X., Wu P. (2019). Multitemporal SAR Image Despeckling Based on a Scattering Covariance Matrix of Image Patch. Sensors.

[26] Paulson C., Zelnio E. The effect of SAR imaging conditions on classification algorithms. Proceedings of the 2014 IEEE Radar Conference (RadarConf16).

